# Lynch Syndrome: Awareness among Medical Students at a United States Medical School

**DOI:** 10.2174/157340412803760667

**Published:** 2012-08

**Authors:** Melissa K Frey, Mollie A Biewald, Michael J Worley, Jolyn S Taylor, Stephanie N Lin, Kevin Holcomb

**Affiliations:** 1 Department of Obstetrics and Gynecology, New York Presbyterian Hospital, Weill Cornell Medical Center, New York, New York 10065, USA; 2 Weill Cornell Medical College, New York Presbyterian Hospital, New York, New York 10065, USA; 3 Division of Gynecologic Oncology, Department of Obstetrics and Gynecology, Brigham and Women’s Hospital, Boston, Massachusetts 02115, USA; 4 Division of Gynecologic Oncology, Department of Obstetrics and Gynecology New York Presbyterian Hospital, Weill Cornell Medical Center, New York New York, 10065, USA

**Keywords:** Hereditary cancer, lynch syndrome, medical education, medical student, screening.

## Abstract

**Introduction:**

Lynch syndrome was first described in the 1950s however until recently it was rarely included in medical school curricula. As a result, many practicing physicians have limited exposure, potentially contributing to significant under diagnosis. As identification of Lynch syndrome prior to malignancy allows for intensified screening, prophylactic surgery and improved patient outcomes, all physicians should be aware of the characteristics of affected families. We aim to determine the overall level of awareness of Lynch syndrome among medical students at an American medical school.

**Methods:**

A voluntary and anonymous questionnaire was delivered to students at an American medical school. The survey instrument assessed the respondent's perceived knowledge regarding the genetics and recommended screening for carriers of Lynch syndrome mutations.

**Results:**

The questionnaire was distributed to the entire student body (405 students) with a response rate of 50%. Fifty-nine percent of students reported that they had learned about Lynch syndrome; 27% of first year students, 44% of second year students; 90% of third year students and 100% of fourth year students. Of the students familiar with Lynch syndrome, the reported knowledge of the underlying genetics was 46%, available genetic screening, 18%, criteria used to screen for the syndrome, 24%, recommendations for colon screening, 31% and recommendations for endometrial cancer screening, 17%.

**Conclusion:**

The majority of medical students surveyed had been exposed to Lynch syndrome and awareness increased over each year of education. Significantly more students were aware of recommendations for colon cancer screening than endometrial cancer screening (32% versus 17%, p = 0.01). Studies of the natural history of Lynch syndrome indicate that affected women are more likely to present with endometrial cancer than colon cancer and while there are no prospective data proving the efficacy of endometrial cancer screening in this high-risk population, the endometrium is easily accessible and can be sampled using simple office techniques. In addition, prophylactic hysterectomy and bilateral salpingo-oophorectomy are reasonable risk reducing interventions for the prevention of both uterine and ovarian cancer. Our findings suggest that increased emphasis must be placed on teaching the gynecologic manifestations of Lynch Syndrome in order to avoid the misconception that it is simply a colon cancer syndrome.

## INTRODUCTION

Lynch syndrome, also known as hereditary nonpolyposis colorectal cancer (HNPCC), is an autosomal dominant hereditary cancer syndrome caused by germline mutations in mismatch repair genes [1]. Lynch mutation carriers have a significantly increased risk of developing cancers of the colon, endometrium, ovary, stomach, hepatobiliary tract, pancreas, small bowel, urinary tract, and central nervous system [2]. The true incidence of Lynch Syndrome remains unknown. Although it has been estimated to be between 1 in 2000 and 1 in 660, this almost certainly represents an underestimate as all current screening methods are less than 100% sensitive, we have only recently started to screen for deletions and most of the available data is based solely on the colon cancer population and does not include endometrial cancers and other Lynch-associated malignancies [3].

Timely identification of Lynch syndrome is critical as it allows for screening that, according to previously published prospective data, can improve patient outcomes [4-6]. However, diagnosis is difficult because, with the exception of the cutaneous stigmata in the Muir-Torre syndrome variant (characterized by sebaceous adenomas, sebaceous carcinomas and keratocanthomas), there are no clinical pathognomonic signs. Instead, diagnosis relies on a detailed cancer family history with attention to malignancies of all anatomic sites through a minimum of three generations by a physician aware of the clinicopathologic features that comprise the syndrome [7]. 

There are two sets of diagnostic criteria used to identify families at risk for Lynch syndrome, the Amsterdam criteria and the Bethesda criteria. The Amsterdam criteria, first published in 1990 by the International Collaborative Group on Hereditary Non-Polyposis Colon Cancer and modified in 1999, suggest that Lynch syndrome should be suspected in kindreds with 1) Three or more relatives with histologically verified Lynch syndrome-associated cancers, one of whom is a first degree relative of the other two and in whom familial adenomatous polyposis has been excluded 2) Lynch syndrome-associated cancers involving at least three generations 3) One or more cancers diagnosed before the age of 50 [8].****The Bethesda criteria, establish by the National Cancer Institute in 1997 and revised in 2004 suggest that tumors from individuals should be tested for microsatellite instability in the following situations 1) Colorectal cancer diagnosed in a patient who is less than 50 years of age 2) Presence of synchronous, metachronous colorectal, or other HNPCC-associated tumors, regardless of age 3) Colorectal cancer with the microsatellite instability-like histology, diagnosed in a patient who is less than 60 years of age 4) Colorectal cancer diagnosed in a patient with one or more first-degree relatives with an HNPCC-related tumor, with one of the cancers being diagnosed under age 50 years 5) Colorectal cancer diagnosed in a patient with two or more first- or second-degree relatives with HNPCC-related tumors, regardless of age [9]. 

Although Lynch Syndrome has been described in the medical literature since the 1950s and much of the genetic etiology uncovered in the early 1990s, many patients remain undiagnosed or have a significant delay in diagnosis, which can have critical implications for affected patients and their family members [10]. One proposed explanation points to medical education. Lynch syndrome is a relatively recently discovered syndrome that, until the past few years, had minimal circulating information on surveillance, management and molecular genetic testing. As a result, the majority of American medical schools did not include Lynch syndrome in student curricula prior to the past decade and therefore many practicing physicians may lack the necessary training to identify this syndrome [7].

Today, with multiple Lynch syndrome mutations identified, accessible genetic screening, growing research and clinical attention paid to medical genetics, the majority of medical students are formally exposed to hereditary cancer syndromes in the preclinical years of medical education [11]. Basic knowledge of Lynch syndrome is important for physicians of any specialty as a thorough family history and genetic testing will increase rates of diagnosis. Furthermore, patients are increasingly curious about their family cancer histories and their personal cancer risk. These questions will not be restricted to oncologists and geneticists. 

Although medical schools are incorporating Lynch syndrome into medical student education and questions are appearing on standardized exams, medical student awareness of this topic has not been formally studied. Our goal was to evaluate the knowledge of Lynch syndrome among first, second, third and fourth year medical students at a single accredited United States medical school and to determine if this knowledge increases over the four years of medical school. Our hypothesis is that with the expanding clinical and research interest in the syndrome and the effort of medical schools to include modules on hereditary cancer syndromes, the majority of medical students will be aware that this syndrome exists and have a basic understanding of the underlying mechanism and clinical implications for affected patients. Furthermore, we hypothesize that a medical student’s knowledge of Lynch syndrome will increase with each year of education.

## METHODS

Approval for this study was received from the institutional review board. A questionnaire was administered to all students in the final three months of the first, second, third and fourth year of medical school at Weill Cornell Medical College, a United States accredited medical school affiliated with a major tertiary care teaching hospital. Internet surveying was not utilized. All students were surveyed between April and June 2011. Participation was voluntary, anonymous and no incentive for questionnaire completion was offered. Students were queried at two separate times; the questionnaire was administered to each class (first year through fourth year) during a lecture and a questionnaire was placed in each student’s medical school mailbox. The questionnaire instrument assessed the perceived knowledge of the medical student regarding the genetics and inheritance patterns of Lynch syndrome, the genetic tests available for Lynch syndrome, the criteria for recommending genetic testing, and the screening modalities recommended for patients with Lynch syndrome. For comparison purposes, the instrument elicited information regarding medical student gender, race and specialty and subspecialty interests.

Data were analyzed using commercially available software (SPSS version 16.0; SPSS, Inc., Chicago, IL). The χ2 and Fisher’s exact tests were used for comparison of proportions. A *p* value of <.05 was considered significant for all tests. The normality of the data was evaluated using normality plots and histograms, with evaluation of skewness and kurtosis. The sample size of 201 participants provided greater than 99.9% power for detecting a difference between medical knowledge among first, second, third and fourth year medical students (omega = 0.61), using a 2-tailed χ2 test with statistical significance defined as *p* <.05. 

## RESULTS

 The student body surveyed consisted of 405 students. 201 questionnaires were completed and returned (response rate 50%). Forty-three percent of respondents were male and 55% female. Fifty-six percent of respondents were non-Hispanic white, 36% Asian, 20% black, 17% other and 14% Hispanic. The breakdown by year in medical school for respondents was as follows: first year 55 students (27%), second year 70 students (35%), third year 41 students (20%) and fourth year 35 students (17%). When asked about potential career interests, 5% of students reported being interested in obstetrics and gynecology, 13% in general surgery and 11% in oncology (Table **1**).

Fifty-nine percent of students reported having had learned about Lynch syndrome and 41% were not familiar with the condition. Of the students who had learned about Lynch syndrome, 46% reported knowledge of the underlying genetic mechanism and pattern of inheritance, 18% reported knowledge of available genetic screening tests, 23% reported knowledge of the criteria used to screen for the syndrome, 32% reported being aware of the recommendations for colon cancer screening and 17% reported being aware of the recommendations for endometrial cancer screening (Table **2**).

**Fig. (1):**
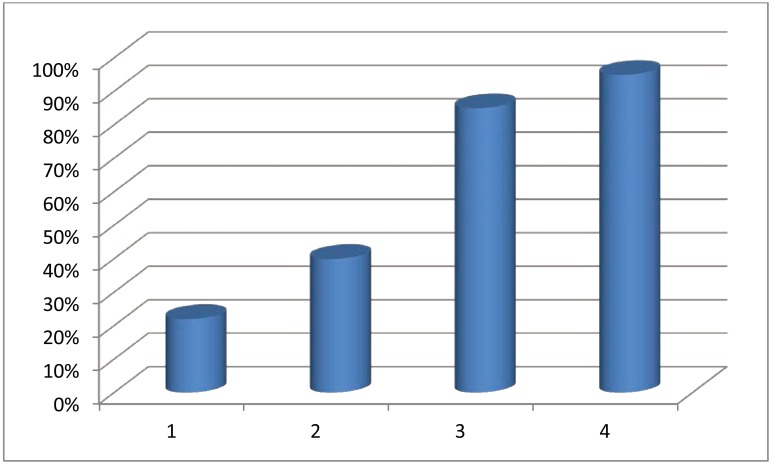
Lynch syndrome awareness among medical students in the first, second, third and fourth year of education.

When evaluated by year in medical school, the results were as follows: 27% of first year students reported having learned about Lynch syndrome, 44% of second year students, 90% of third year students and 100% of fourth year students (Fig. **1**). For all areas of perceived knowledge assessed (genetics/inheritance, genetic screening, criteria for recommending genetic screening and recommendations for colon and endometrial cancer screening), student reported knowledge significantly increased between the first year of medical school and the fourth year of medical school (Table **3**).

There were no differences in perceived Lynch syndrome knowledge based on student gender, race or interest in obstetrics and gynecology, general surgery, or oncology. Only 11 students of the 201 medical students reported having been involved in the care of a patient with Lynch syndrome.

## DISCUSSION

With the sequencing of the human genome and significant advances in biotechnology over the past few years, the complexity and breadth of medical genetics are growing rapidly. Surprisingly, while genetic screening, diagnosis and treatment are becoming integrated into all areas of medicine, there has not been an equivalent rise in the number of physicians choosing to practice the specialty of medical genetics. There have been only 1,326 new physician geneticists to receive board certification between 1982 and 2009, representing less than 0.3 percent of the physicians in the United States. The number of physicians pursuing this field has remained flat over the past 15 years [12]. Further limiting access to genetics consultations, physician geneticists traditionally have devoted only 50 percent of their time to direct patient care and practice trends favor specialization in rare diseases as opposed to a general genetics practice [13]. As a result the number of genetics professionals certified by the American Board of Medical Genetics cannot meet the growing demand for genetics services [14]. 

Recognizing the emerging importance of genetics in medicine, the Association of American Medical Colleges issued a report in 2004 entitled “Contemporary Issues in Medicine: Genetics Education” championing the importance of genetics education and outlining core competencies in genetics that all medical students should attain by the end of their training [15]. Previous studies evaluating physician knowledge of human genetics found that both formal course work in genetics and more recent year of graduation from medical school were significantly associated with mean total knowledge scores [16]. Evidence for a direct effect of such courses on knowledge comes from a study in which time devoted to teaching genetics to medical students was a predictor of the students’ ability to correctly answer genetics questions on the National Board of Medical Examiners Examination [17]. 

At Weill Cornell Medical College there is a formal course in human genetics during the first year of medical school. During this course students receive one lecture on hereditary cancer genetics, which includes information on Lynch syndrome. However, based on the results of this questionnaire, it appears that much of the memorable exposure emerges during the second year as students learn pathophysiology and throughout the clinical clerkships in the third and fourth years. Our questionnaire suggests that exposure to Lynch syndrome increases with each year of medical school. Furthermore, knowledge regarding the inheritance patterns, genetic screening, criteria for recommending genetic screening and recommendations for colon and endometrial screening also each increase incrementally as students advance through medical school. Interestingly, all fourth year students surveyed reported having learned about the syndrome despite the minority having participating in the care of a patient with this disease. This likely reflects emphasis on hereditary cancer syndromes among medical instructors and increased awareness and discussion of this topic on the hospital wards. However, the acquisition of knowledge may also be affected by student preparation for the United States Medical Licensing Examination (USMLE). Students at Weill Cornell Medical College typically take step one of the exam immediately following the second year of medical school and step two during the fourth year.

It is concerning, however, that significantly more students were aware of recommendations for colon cancer screening than endometrial cancer screening (32% versus 17%, *p* = 0.01). Studies of the natural history of Lynch syndrome indicate that affected women are more likely to present with endometrial cancer than colon cancer [1, 14]. While there are no prospective data proving the efficacy of endometrial cancer screening in this high-risk population, the endometrium is easily accessible and can be sampled using simple office techniques. In addition, prophylactic hysterectomy and bilateral salpingo-oophorectomy are reasonable risk reducing interventions for the prevention of both uterine and ovarian cancer [18]. Our findings suggest that increased emphasis must be placed on teaching the gynecologic manifestations of Lynch Syndrome in order to avoid the misconception that it is simply a colon cancer syndrome. 

The rapid growth of the biology, clinical implications and patient interest in medical genetics has been particularly notable in the field of hereditary cancer genetics. Many patients are learning from the media, pharmaceutical companies and commercial DNA laboratories about hereditary cancer syndromes. Patients look to their physicians for answers regarding cancer risk, screening and surveillance. When confronted with any patient with a complex family cancer pedigree, a physician must be aware of the basic clinicopathologic features of Lynch syndrome in order to appropriately refer patients for genetic testing and counseling and answer complex patient questions. This identification is crucial in patients affected with Lynch syndrome as diagnosis allows for patients to initiate aggressive cancer screening [4, 5]. 

Our questionnaire reveals that medical students at a single accredited United States medical school are learning about Lynch syndrome. Knowledge seems to come from both formal coursework during the first year and then informal exposure during the second through fourth years. The acquisition of knowledge appears not to be related to the gender, race or career aspirations of the medical student, suggesting that this topic emerges routinely during the course of medical education. However, this study was not adequately powered to determine the student characteristics associated with Lynch syndrome knowledge. While the response rate to the questionnaire tool was only 50%, there was no statistically significant difference between the gender, race and career aspirations of the 201 students included in the sample and the entire student body. These results are promising given the importance of physician knowledge for the ability to diagnose Lynch syndrome and a current climate of increased patient awareness, fear and interest in learning about genetic risk for the development of hereditary cancers. Increased emphasis, however, must be given to the gynecologic manifestations of Lynch syndrome and risk-reducing options for gynecologic cancer prevention. 

## Figures and Tables

**Table 1.** Medical Student Characteristics

**Gender (n, %)**	
Male	87 (43.3)
Female	110 (54.7)
**Race (n, %)**	
Hispanic	14 (7.0)
Asian	36 (17.9)
Non-Hispanic White	112 (55.7)
Black	20 (10.0)
Other	17 (8.5)
**Year in Medical School (n, %)**	
First	55 (27.4)
Second	70 (34.8)
Third	41 (20.4)
Fourth	35 (17.4)
**Interested in Obstetrics and Gynecology (n, %)**	
No	188 (93.5)
Yes	10 (5.0)
**Interested in General Surgery (n, %)**	
No	174 (86.6)
Yes	26 (12.9)
**Interested in Oncology (n, %)**	
No	178 (88.6)
Yes	22 (10.9)

**Table 2.** Knowledge of Lynch Syndrome among Students who Reported having Learned about the Disease

**Learned about Lynch Syndrome (n, %)**	
No	83 (41.3)
Yes	118 (58.7)
**Involved in a Lynch Syndrome Case (n, %)**	
No	106 (89.8)
Yes	11 (9.3)
**Aware of Genetics/Inheritance (n, %)**	
No	64 (54.2)
Yes	54 (45.8)
**Aware of Genetic Screening Available (n, %)**	
No	97 (82.2)
Yes	21 (17.8)
**Aware of Criteria for Screening (n, %)**	
No	90 (76.3)
Yes	28 (23.7)
**Aware of Recommendations for Colon Screening (n, %)**	
No	81 (68.6)
Yes	37 (31.4)
**Aware of Recommendations for Endometrial Screening (n, %)**	
No	98 (83.1)
Yes	20 (16.95)

**Table 3.** Knowledge of Lynch Syndrome by Year in Medical School

	First Year (n, %)		Second Year (n, %)		Third Year (n, %)		Fourth year (n, %)		*P* value
	No	Yes	No	Yes	No	Yes	No	Yes	
**Learned about Lynch syndrome**	40 (72.7)	15 (27.3)	39 (55.7)	31 (44.3)	4 (9.8)	37 (90.2)	0 (0)	35 (100)	<0.001
**Involved in a Lynch Syndrome Case**	54 (98.2)	1 (1.8)	70 (100)	0 (0)	37 (90.2)	4 (9.8)	28 (80)	6 (17.1)	0.002
**Aware of Genetics/Inheritance**	53 (96.4)	2 (3.6)	44 (62.9)	26 (37.1)	28 (68.3)	12 (31.7)	21 (60)	14 (40)	<0.001
**Aware of Genetic Screening Available**	55 (100)	0 (0)	61 (87.1)	9 (12.9)	35 (85.4)	6 (14.6)	29 (82.9)	6 (17.1)	<0.001
**Aware of Criteria for Screening**	55 (100)	0 (0)	66 (94.3)	4 (5.7)	33 (80.5)	8 (19.5)	27 (77.1)	8 (22.9)	<0.001
**Aware of Recommendations for Colon Screening**	55 (100)	0 (0)	56 (80)	14 (20)	35 (85.4)	6 (14.6)	17 (48.6)	18 (51.4)	<0.001
**Aware of Recommendations for Endometrial Screening**	55 (100)	0 (0)	63 (90)	7 (10)	38 (92.7)	3 (7.3)	25 (71.4)	10 (28.6)	<0.001

## ABBREVIATIONS

HNPCC: = Hereditary nonpolyposis colorectal cancer
USMLE: = United States Medical Licensing Examination
